# Supplementary material for: Dietary total antioxidant capacity of Moroccan Type 2 Diabetes Mellitus patients
Source: PLoS One. 2024 Apr 16;19(4):e0301805. doi: 10.1371/journal.pone.0301805 (PMC11020496; doi:10.1371/journal.pone.0301805)
Supplement: Attachment — Submitted filename: Response to Reviewers.docx [file pone.0301805.s001.docx]

Faculty of Sciences

Abdelmalek Essaadi University

Laboratory of Applied Biology and Pathology (UAE/U24FS)

Tetouan, MOROCCO	January 08^th^, 2024

[najia.frakchi@gmail.com](mailto:najia.frakchi@gmail.com)

Dear Editors

We thank the academic editor and reviewers for their generous comments on the submitted manuscript and have have edited the manuscript to address their concerns.

We believe that the manuscript is now suitable for publication in PLoS ONE journal.

El Frakchi Najia

PhD researcher

On behalf of all authors.

**Request**

We noticed that this work is related to the following submission of yours currently under review: PONE-D-23-22223 (Association of dietary total antioxidant capacity to general and abdominal obesity in patients with Type 2 Diabetes Mellitus in Northern Morocco)

Upon submission, authors must confirm that the manuscript, or any related manuscript, is not currently under consideration or accepted elsewhere.

In response to your request, I would like to inform you that I have submitted simultaneously to PLOS ONE journal two original research articles that have been written as an independent paper for publication.

The aforementioned manuscripts were the fruit of a survey carried out on the same clinic population of Moroccan patients with T2DM. Although the same methodology was used for data collection and assessment of dietary intake including dietary TAC assessment, the objective of each manuscript was different.

In PONE-D-23-22322 manuscript, we aimed to bring to light new information about the daily intake of antioxidants among patients with T2DM on the basis of the dietary antioxidant index since it is the first study assessing dietary TAC in Moroccan country. The contribution of common food eaten in Morocco to total antioxidant capacity intake was also examined.

In PONE-D-23-22223 manuscript, we aimed to explore the relationship between dietary TAC of these patients and obesity-related features regarding the importance of obesity in this clinic population as a common comorbidity of type 2 diabetes and one of the most modifiable risk factors for preventing other comorbid conditions, such as cardiovascular disease.

We think so that it is desirable to publish separately the above works and not to present the full data within a single manuscript since each one deals with an objective. Also, that may help to provide more easily understandable information.

We declare that the manuscript is original, has not been published before, and is not currently under consideration or accepted elsewhere.

**Reviewer 1:**

The authors should consider the following suggestions:

1. Edit to improve the quality of Figure 1 by showing the line axes and also remove the gridlines.

2. Include a section on Conclusion in the Manuscript

3. It would be helpful to have a control (perhaps non diabetic patient) in this investigation to compare.

The quality of Figure 1 was improved as recommended and conclusion section was included as well.

Agreed. Further case-control designed investigation may be considered thereafter.

**Reviewer 2:**

The authors should clearly state the primary outcome and secondary outcome of the study. The sampling methodology and the research design needs to be clearly spelt out. The inclusion and exclusion criteria are not stated clearly. The statistical treatment to the antioxidant value data with nutrients and food groups is adequate but it does not states clearly discusses the connect between Dietary Antioxidant intake and Type 2 diabetes.

I carefully rewrite the study design and population section with the intent to meet the aforementioned requirements.

I have also added a short sentence to discuss the connect between Dietary Antioxidant intake and Type 2 diabetes through presented data on dietary intake according to dietary antioxidant capacity.
